# Factors associated with institutional delivery in south Asian countries: evidence from five recent demographic and health surveys

**DOI:** 10.1007/s43999-025-00071-3

**Published:** 2025-08-01

**Authors:** Sifat Muntaha Soni, Md. Ismail Hossain, Salma Akter, Shahjadi Ireen, Shuvongkar Sarkar, Shahanaj Parvin, Mansura Begum, Rebeka Sultana

**Affiliations:** 1 Department of Statistics, Islamic University, Kustia, Bangladesh; 2 Department of Mathematics and Natural Sciences, BRAC University, Dhaka, 1212 Bangladesh; 3 Department of Statistics, Jagannath University, Dhaka, Bangladesh

**Keywords:** Institutional delivery, Socio-economic and demographic factors, Maternal mortality, Antenatal visit

## Abstract

**Background:**

Maternal and infant mortality is a major public health concern especially in South Asian nations. A significant proportion of mothers and infant died as a result of complications during birth. The delivery of healthcare facilities plays key role to lowering these mortality rates. The present study aimed to explore the prevalence of institutional delivery and its determinants in five South Asian countries.

**Methods:**

Data were extracted from five South Asian countries latest demographic and health survey data, including Afghanistan (2015), Bangladesh (2017–18), Nepal (2016), Myanmar (2015–16), and Pakistan (2017–18), all of which were pooled for the present study. A total of 38,975 women were included in this study after data handling. A multivariate binary logistic regression model was performed to identify the factors influencing institutional delivery.

**Results:**

More than half of all deliveries among the women were reported as occurring in a medical facility. The proportion of institutional deliveries was highest in Pakistan (68.80%), and lowest in Myanmar (40.60%). This study found that women who give birth at after 20 years’ age had 1.25 times higher chance of getting healthy facility during delivery (OR 1.25, [1.19, 1.32]). The odds of institutional delivery were 2.18 times higher for highly educated women (OR 2.18, [1.89, 2.52]) and 2.88 times higher for rich women (OR 2.88, [2.70, 3.07]). The likelihood of getting his wife delivered in a hospital increased with the husband’s education level. Women who accessed by any media showed 33% higher chance of getting healthy facility during child birth. Women who did not obtain ANC from a skilled provider had a reduced likelihood of selecting healthcare facility delivery by 71% (OR 0.29, [0.28, 0.31]) compared to women who did. Women who didn’t take any health care decision by-self had 16% lower chance of getting institutional delivery facility than others. Most importantly, rural area in south Asian countries presented lower odds of receiving healthy facility during delivery (OR 0.63, [0.59, 0.68]).

**Conclusions:**

In conclusion, improving maternal health among South Asian countries requires addressing both individual and community-level factors. Women with higher education, better socioeconomic status, media exposure, and access to prenatal care are more likely to utilize medical services. Strengthening evidence-based health policies and ensuring strong leadership can enhance women’s quality of life through better access to health care.

## Introduction

Despite a dramatic decline in maternal deaths [1], 800 women still died every day in 2020 from preventable causes related to pregnancy and childbirth—nearly one every two minutes [2]. In lower-middle-income countries, maternal deaths accounted for the majority of the total [3]. More than 80% of global maternal fatalities in 2020 occurred in Sub-Saharan Africa and Southern Asia, with Sub-Saharan Africa alone contributing nearly 70% of maternal mortality [2].

The leading causes of maternal deaths are severe bleeding and infections after childbirth, high blood pressure during pregnancy, and unsafe abortions [4]. Prior studies have revealed that a third of all antepartum causes, including intrapartum and postpartum hemorrhage, are linked to risky home delivery practices [5, 6]. One of the most crucial and effective measures to reduce maternal mortality is the use of institutional delivery services. By ensuring safe delivery and minimizing complications—including actual and potential deaths—maternal and fetal survival rates can be significantly improved [7]. However, it is vital that deliveries are conducted by trained medical professionals who can recognize signs and symptoms of complications [8].

Although significant progress has been made in South Asia over the past 20 years in increasing deliveries at medical facilities, the rate remains low compared to other regions. The primary barriers that prevent women from accessing or seeking health care during pregnancy and childbirth include inadequate services, poverty, distance, cultural practices, and lack of information [9, 10]. Women who give birth at home in South Asia are more likely to be exposed to hazardous and unhygienic conditions, placing both maternal and newborn lives at risk [11].

According to a study, Myanmar has the second-lowest rate of institutional deliveries among Southeast Asian nations, with over one-third of all births occurring in medical facilities [12]. In Bangladesh, home deliveries are common, with only about half of live births taking place in hospitals [13]. Thus, complications during delivery remain one of the leading causes of maternal mortality in Bangladesh. A study conducted in rural Bangladesh found that one-third of women experienced delivery-related problems during their most recent birth [14]. Nearly half of women in Afghanistan gave birth outside of health facilities. In Nepal, the institutional delivery rate among newlyweds was 46% [15], and another study found that nearly two out of every five maternal deaths occur at home [6]. Similar statistics are observed in Pakistan, where 71% of urban and 45% of rural women deliver in medical facilities [16].

Despite the numerous benefits of institutional delivery, it is still not widely adopted in several LMICs in the South Asian region. South Asian nations have implemented various prenatal care packages to promote institutional delivery. However, to the best of our knowledge, no comprehensive research has been conducted on this issue across South Asian countries. Therefore, the objectives of this study were to determine the prevalence of institutional delivery among women aged 15 to 49 in five South Asian countries, examine the associations between sociodemographic and behavioral characteristics and institutional delivery, and identify the risk factors influencing institutional delivery. The analysis utilized pooled data from the Demographic and Health Surveys (DHS) to address these objectives.

## Methods and materials

### Data sources

The analysis of this study was done using five South Asian Demographic and Health Survey data sets, which was a large-scale, nationally representative cross-sectional study. The Demographic and Health Surveys (DHS) Program collected and disseminated information on population and health in developing nations. The data retrieved from the 2015 Afghanistan Demographic and Health Survey (AfDHS), 2017-18 Bangladesh Demographic and Health Survey (BDHS), 2016 Nepal Demographic and Health Survey (NDHS), 2015-16 Myanmar Demographic and Health Survey, and 2017-18 Pakistan Demographic and Health Survey (PDHS). The DHS program is carried out by ICF, which also offers technical support. The United States Agency for International Development (USAID) provided funding for the survey.

### Sample design

The DHS survey employed a two-stage stratified sampling technique within the sampling units. Current Demographic and Health Surveys (DHS) data were combined for this study. Data were restricted to the DHS from five South Asian nations: Bangladesh (2017–18), Pakistan (2017–2018), Nepal (2016), Afghanistan (2015), and Myanmar (2015–2016). The main justification for choosing these five nations was the fact that information on some of the relevant sociodemographic and behavioral traits was only accessible in these nations. The total sample size for the present study was 38,975.

### Dependent variable

The binary outcome variable was institutional delivery, which was dichotomously categorized as “Yes” or “No”. If a delivery took place in a hospital, public or private, it was classified as facility-based; otherwise, it was classified as “No.”

### Independent variables

A set of categorical explanatory variables was selected to fit the two individual regression models. In Model 1, we only included the origin country as an independent variable. Based on the several studies, twelve explanatory variables were considered independent variables in Model 2, namely women’s age at first birth, women’s education [17], husband’s education [18], wealth status [18], mass media access [19], ANC by skilled providers [20], 4 + ANC visits [21], early ANC intention [22], decision-making on own health [22], child ever born [22], residence [22], and country of origin (Bangladesh, Pakistan, Nepal, Afghanistan, Myanmar).

### Statistical analysis

This study used descriptive analysis to gain a general understanding of the sample’s characteristics. The chi-square test of independence was employed to assess the relationship between institutional delivery and selected covariates. In the multivariate setting, two distinct binary logistic regression models were fitted. The impact of the nation on institutional delivery is examined through Model 1. Model 2 investigated the influence of particular sociodemographic and behavioral characteristics on institutional delivery. Data processing was done using SPSS version 25 (IBM Corporation, Armonk, NY, USA), and statistical analysis was done using R Project for Statistical Computing version 4.0.0.

## Results

Table 1 represents the sociodemographic characteristics of women in South Asia. The data reveals that more than half of the mothers gave birth in their adolescent period (under 20 years old). In terms of education, the highest proportion of the women were uneducated, followed by primary, secondary, and higher. Majority of women’s husband had no formal education (39.5%). Furthermore, Table 1 demonstrates that the proportion of the variable was higher among women who were poor (41.4%), had access to the media (53.5%), had ANC performed by a skilled provider (71.5%), had less than 4 ANC visits (63.6%), and intended to have an early ANC (60.3%). Nearly a third quarter of women resided in rural areas. Women from Afghanistan make up more than half of the population, followed by Pakistan, Bangladesh, Nepal, and Myanmar. The majority of the women were delivered in a medical facility.

**Table 1:** Distribution of South Asian women’s sociodemographic characteristics

Variables	Frequency (*n* = 38975)	Percentage
**Women age at first birth**		
< 20 years	20,246	51.9
$\:\ge\:$ 20 years	18,729	48.1
**Women education**		
No education	21,652	55.6
Primary	6494	16.7
Secondary	7812	20.3
Higher	3018	7.7
**Husband education**		
No education	15,395	39.5
Primary	7901	20.3
Secondary	11,257	28.9
Higher	4422	11.3
**Wealth status**		
Poor	16,128	41.4
Middle	7848	20.1
Rich	14,998	38.5
**Mass media access**		
Not exposed	18,112	46.5
Exposed	20,863	53.5
**ANC by skilled provider**		
Yes	27,829	71.5
No	11,100	28.5
**4 + ANC visit**		
Yes	14,193	36.4
No	24,782	63.6
**Early ANC intention**		
Yes	15,468	39.7
No	23,507	60.3
**Decision making**		
Self or Jointly	25,798	66.2
Others	13,177	33.8
**Child ever born**		
1	8971	23.0
2–3	14,682	37.7
4+	15,322	39.3
**Residence**		
Urban	11,225	28.8
Rural	27,750	71.2
**Country**		
Pakistan	6711	17.2
Afghanistan	19,632	50.4
Bangladesh	5051	13.0
Myanmar	3583	9.2
Nepal	3998	10.3
**Health facility delivery**		
Yes	21,012	53.9
No	17,963	46.1

The relationships between sociodemographic factors and the usage of a health facility during childbirth showed in Table 2. In this analysis, all of the covariates were significantly (*P* < 0.05) correlated with institutional delivery. The percentage of women who used a health facility during delivery was found to be higher for older women ( $\:\ge\:$20 years) (approximately 60%), higher educated women (86.7%), husbands with higher levels of education (81.3%), wealthy women (approximately 76.5%), exposed to media (65.4%), skilled providers performing ANC (65.1%), having more than four ANC visits (73.4%), early ANC intention (69%), giving birth to their first child (67.9%), and urban women (75.4%).

**Table 2:** Association between South Asian women’s socioeconomic characteristics and institutional delivery

Variables	Institutional delivery		$\:{\varvec{\chi\:}}^{2}$ value (*p*-value)
	Yes (%)	No (%)	
**Women age at first birth**			
< 20 years	47.8	52.2	623.73 (< 0.001)
$\:\ge\:$ 20 years	60.5	39.5	
**Women education**			
No education	45.5	54.5	2599.01 (< 0.001)
Primary	50.1	49.9	
Secondary	67.4	32.6	
Higher	87.6	12.4	
**Husband education**			
No education	41.1	58.9	2972.92 (< 0.001)
Primary	48.4	51.6	
Secondary	64.6	35.4	
Higher	81.3	18.7	
**Wealth status**			
Poor	34.7	65.3	5379.85 (< 0.001)
Middle	51.0	51.0	
Rich	76.1	76.1	
**Mass media access**			
Not exposed	40.7	59.3	2373.50 (< 0.001)
Exposed	65.4	34.6	
**ANC by skilled provider**			
Yes	65.1	34.9	4893.07 (< 0.001)
No	26.0	74.0	
**4 + ANC visit**			
Yes	73.4	26.6	3402.54 (< 0.001)
No	42.8	57.2	
**Early ANC intention**			
Yes	69.0	31.0	2343.61 (< 0.001)
No	44.0	56.0	
**Decision making**			
Self or Jointly	55.6	44.4	86.08 (< 0.001)
Others	50.6	49.4	
**Child ever born**			
1	67.9	32.1	1146.40 (< 0.001)
2–3	54.1	45.9	
4+	45.5	54.5	
**Residence**			
Urban	75.4	24.6	2940.45 (< 0.001)
Rural	45.2	54.8	

**Fig. 1:**
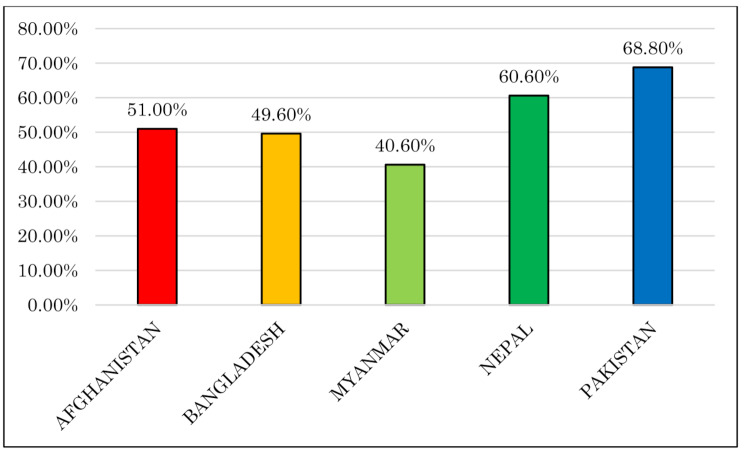
Proportion of institutional delivery across the country

Figure 1 shows the frequency of health facility delivery in five countries in South Asia. Institutional delivery was most prevalent in Pakistan (around two-thirds), whereas it was least prevalent in Myanmar (almost two-fifths).

Table 3 presents the factors influencing institutional delivery among women in five countries in South Asia. The unadjusted effect of the country on health facility delivery was estimated in Model 1. From this model, it was found that Pakistan had the highest odds (OR 2.24, [2.08–2.42]) compared to Bangladesh, followed by Nepal (OR 1.56, [1.44–1.70]), and Myanmar (OR 0.69, [0.64–0.76]). In Model 2, after adjusting the socio-demographic characteristics, an almost similar result was discovered for the countries except Afghanistan (OR 3.76, [3.41, 4.14]), which was the second highest user of health care facilities during delivery compared to Bangladesh. In Pakistan, institutional deliveries were almost four times more probable than in Bangladesh (OR 4.07, [3.67, 4.52]), whereas in Nepal AOR is 1.49, [1.34, 1.66]). However, all the covariates included in Model 2 were significantly associated with institutional delivery. Older women (age above 20) were significantly 25% more likely to receive health care facilities during delivery (OR 1.25, [1.19–1.32]) compared to young women (age below 20). Chance of health facility delivery increased with the increase of education level. Higher-educated women had more than twice the odds of using institutions for delivery (OR 2.18, [1.89, 2.52]) than uneducated women. In comparison to women with an uneducated husband, women whose husband had a higher education had 83% more chance of using a health facility during delivery (OR 1.83, [1.65–2.03]). Additionally, Rich women were 88% more likely than poor women to give birth at a hospital (OR 2.88 [2.70, 3.07]), whereas middle-class women were 54% more likely (OR 1.54 [1.45, 1.64]). The use of medical care for childbirth was impacted by the mass media; exposed women had a 33% higher likelihood of choosing institutional delivery (OR 1.33, [1.26–1.40]) than non-exposers. Women who did not obtain ANC from a skilled provider had a reduced likelihood of selecting healthcare facility delivery by 71% (OR 0.29, [0.28, 0.31]) compared to women who did. More than four ANC visits by a woman increased her likelihood of institutional delivery by 50%. Women who were made to make decisions by others were 16% (OR 0.84, [0.80, 0.89]) less likely to deliver in a health facility than women who made to make decisions by own or jointly. Interestingly, as the number of children increased, the likelihood of choosing institutional delivery declined. Rural women were 37% less likely to deliver in a medical setting than their counterparts.

**Table 3:** Multivariate logistic regression analyses for the identification of the determinants influencing institutional delivery among women in five South Asian countries

Variables	Unadjusted Odds Ratio (95% CI)	*p*-value	Adjusted Odds Ratio (95% CI)	*p*-value
**Country**				
Bangladesh	Ref.		Ref.	
Pakistan	2.24 (2.08, 2.42)	< 0.001	4.07 (3.67, 4.52)	< 0.001
Afghanistan	1.06 (1.00, 1.12)	0.08	3.76 (3.41, 4.14)	< 0.001
Myanmar	0.69 (0.64, 0.76)	< 0.001	0.64 (0.57, 0.71)	< 0.001
Nepal	1.56 (1.44, 1.70)	< 0.001	1.49 (1.34, 1.66)	< 0.001
**Women’s age at first birth**				
< 20 years			Ref.	
$\:\ge\:$ 20 years			1.25 (1.19, 1.32)	< 0.001
**Women education**				
No education			Ref.	
Primary			1.23 (1.14, 1.33)	< 0.001
Secondary			1.48 (1.36, 1.61)	< 0.001
Higher			2.18 (1.89, 2.52)	< 0.001
**Husband education**				
No education			Ref.	
Primary			1.22 (1.14, 1.30)	< 0.001
Secondary			1.40 (1.31, 1.50)	< 0.001
Higher			1.83 (1.65, 2.03)	< 0.001
**Wealth status**				
Poor			Ref.	
Middle			1.54 (1.45, 1.64)	< 0.001
Rich			2.88 (2.70, 3.07)	< 0.001
**Mass media access**				
Not exposed			Ref.	
Exposed			1.33 (1.26, 1.40)	< 0.001
**ANC by skilled provider**				
Yes			Ref.	
No			0.29 (0.28, 0.31)	< 0.001
**4 + ANC visit**				
Yes			Ref.	
No			0.50 (0.47, 0.54)	< 0.001
**Early ANC intention**				
Yes			Ref.	
No			0.95 (0.89, 1.02)	0.60
**Decision making**				
Self or Jointly			Ref.	
Others			0.84 (0.80, 0.89)	< 0.001
**Child ever born**				
1			Ref.	
2–3			0.60 (0.56, 0.64)	< 0.001
4+			0.49 (0.46, 0.53)	< 0.001
**Residence**				
Urban			Ref.	
Rural			0.63 (0.59, 0.68)	< 0.001

## Discussion

This study examined the prevalence of institutional delivery and its contributing aspects in Bangladesh, Nepal, Pakistan, Afghanistan, and Myanmar, five South Asian nations. This study found that the rate of health facility delivery was higher among highly educated women. Higher educated women are more likely to be aware of and knowledgeable about health-related issues. This result was backed up by prior studies [23–27]. Previous research showed that wealthy mothers were more likely to deliver their babies in a hospital [28, 29]. The same outcome came from this study. One of the major obstacles to the delivery of health care is the poor’s inability to afford the costs of such services. The likelihood of giving birth in a hospital was lower for rural women than for urban ones. This finding was in line with the previous study of Bangladesh [30].

This study also demonstrated that the usage of healthcare services during delivery was significantly influenced by the media. It was backed up by earlier research [30, 31]. Another study, however, produced inconsistent results [23]. Women who received care from qualified staff were more likely to give birth in a safe setting. This result is similar to studies conducted in Africa that concluded that having better prenatal care increases the risk of having a baby in a hospital [32]. In this study, we also found that women who had more than four ANC visits were more likely to use health facility during delivery. Similar findings were found in previous research [33, 34]. Prior studies showed that women with low parity could be more motivated to deliver in health facilities [7, 23, 35]. This study also found that women with high parities (more than 1) were less likely to have health facility deliveries. The reason is that family members are treated more seriously at the first delivery. This study found an association between a woman’s age at her first birth and delivery in a medical facility. The consistent outcome was obtained from a study conducted in sub-Saharan Africa [5].

Women with highly educated husbands were more likely to deliver in a health facility compared to their counterparts. Educated husbands are more concerned about the health of their wives and children and therefore ensure institutional delivery. A similar result was found in the previous study in Bhutan [36]. According to this study, women who made decisions on their own or jointly were more likely to use a health facility during delivery than those who made decisions with others. This finding is consistent with the previous studies [37, 38].

The drawback of this study is that it is based on DHS data. The sample sizes at the country level are frequently not large enough. Our research only includes deliveries that result in live births since DHS data does not contain pregnancy outcomes (and their location) other than live births.

## Conclusions

One of the main issues hindering the attainment of SDG 3 in many developing nations is the lack of access to health facilities. The present study showed that women with higher education, good socio-economic conditions, media exposure, age at first birth, and husband education were the contributing factors to having a health facility delivery. In these five South Asian nations, there are still significant obstacles due to a lack of awareness regarding the significance of receiving delivery care from a qualified practitioner. The general public’s knowledge would be significantly raised by the media. The quantity of media exposure should be increased through the initiatives of policymakers. Low parity influences the utilization of healthcare facility delivery. The emphasis on low-parity women should be strengthened even though research and policy are trending in that direction. It is vital to enhance access to health facility delivery, particularly in rural areas. The inadequate use of healthcare facilities during delivery was mostly caused by women’s socioeconomic situation and level of education. Therefore, when formulating health policies and plans, the governments of these five countries should place more focus on these two criteria.

## Data Availability

In this study, we used data from Demographic Health Survey (DHS), which is available from https://dhsprogram.com/data/available-datasets.cfm.
